# LPS induces cardiomyocyte injury through calcium-sensing receptor

**DOI:** 10.1007/s11010-013-1637-3

**Published:** 2013-04-08

**Authors:** Hong-yu Wang, Xue-yan Liu, Gan Han, Zhu-ying Wang, Xiao-xie Li, Zhi-mei Jiang, Chun-ming Jiang

**Affiliations:** 1 Department of Neonatology, The First Clinical Hospital of Harbin Medical University, Harbin, 150001 China; 2 Children’s Rehabilitation Laboratory of Jiamusi University, Jiamusi, 154002 China

**Keywords:** Calcium-sensing receptor, Cardiomyocyte, Lipopolysaccharide, TNF-α, IL-6, Apoptosis

## Abstract

Calcium-sensing receptor (CaSR) belongs to the family C of G-protein coupled receptors. We have previously demonstrated that CaSR could induce apoptosis of cultured neonatal rat ventricular cardiomyocytes in simulated ischemia/reperfusion. It remains unknown whether the CaSR has function in lipopolysaccharide (LPS)-induced myocardial injure. The aim of this study was to investigate whether the CaSR plays a role in LPS-induced myocardial injury. Cultured neonatal rat cardiomyocytes were treated with LPS, with or without pretreatment with the CaSR-specific agonist gadolinium chloride (GdCl_3_) or the CaSR-specific antagonist NPS2390. Release of TNF-α and IL-6 from cardiomyocytes was observed. Levels of malonaldehyde (MDA), lactate dehydrogenase (LDH), and activity of superoxide dismutase (SOD) were measured. In addition, apoptosis of the cardiomyocytes, [Ca^2+^]_i_ and level of CaSR expression were determined. The results showed that LPS increased cardiomyocytes apoptosis, [Ca^2+^]_i_, MDA, LDH, TNF-α, IL-6 release, and CaSR protein expression. Compared with LPS treatment alone, pretreatment with GdCl_3_ further increased apoptosis of cardiomyocytes, MDA, LDH, TNF-α, IL-6 release, [Ca^2+^]_i_, and the expression of the CaSR protein. Conversely, pretreatment with NPS2390 decreased apoptosis of cardiomyocytes, MDA, LDH, TNF-α, IL-6 release, [Ca^2+^]_i_ and the expression of the CaSR protein. These results demonstrate that LPS could induce cardiomyocyte injury. Moreover, LPS-induced cardiomyocyte injury was related to CaSR-mediated cardiomyocytes apoptosis, TNF-α, IL-6 release, and increase of intracellular calcium.

## Introduction

Sepsis is a common complication in neonatal intensive care units. The incidence of neonatal sepsis is 1–5 per 1,000 live births, and its mortality rate is 5–20 % [1, 2]. The incidence of sepsis and sepsis-related deaths is increasing by 1.5 % per year. One cause of death among affected patients is severe hypotension associated with a decrease in cardiac output [3, 4]. Currently, accumulating evidence has indicated that myocardial depression is a common feature of sepsis in both neonates and experimental models of lipopolysaccharide (LPS)-induced endotoxemia [5–7]. In this way, novel therapies that can be used to prevent or treat this devastating disease are urgently required.

Intracellular calcium, a secondary messenger, plays a key role in various physiological processes. Many studies have shown that extracellular calcium can act as a first messenger through the calcium-sensing receptor (CaSR) in various cells [8]. CaSR belongs to the family C of G-protein coupled receptors. It was first cloned in 1993 from bovine parathyroid gland by Brown [9]. CaSR is important in maintaining and regulating mineral ion homeostasis [10]. Wang et al. [11, 12] found CaSR to be functionally expressed in the cardiovascular system. Binding of extracellular Ca^2+^ or other CaSR agonists, and the activation of the receptor trigger many intracellular signaling events [13, 14]. CaSR is involved in acute myocardial infarction, in the progress of diabetic cardiomyopathy, and in cyclosporin A-induced cardiomyocyte apoptosis in rats [15–17].

Calcium-sensing receptor can induce apoptosis among cultured neonatal rat ventricular cardiomyocytes in simulated ischemia/reperfusion [18–20]. It remains unknown whether the CaSR plays a part in LPS-induced myocardial injury. In this way, the present study was designed to examine the possible effects of CaSR in LPS-induced inflammation and heart injury and to provide direct pharmacological evidence to determine whether CaSR is involved in this process.

## Materials and methods

The study was approved by the Institutional Animal Research Committee and all animals received humane care in compliance with the Guide for the Care and Use of Laboratory Animals published by the National Institute of Health (NIH publication 86–23, revised 1986).

### Animals

Sprague–Dawley rats, 1–2 days old, were obtained from the laboratory animal center of Harbin Medical University.

### Materials

Lipopolysaccharide (LPS) from *Escherichia coli* serotype 055:B5, GdCl_3_ (product number 450855) and quinoxaline-2-carboxylic acid adamantan-1-ylamide (NPS2390, product number N4786) were purchased from Sigma-Aldrich (St Louis, MO, U.S.). Anti-CaSR antibody was purchased from Alpha Diagnostic International (San Antonio, TX, U.S.). Quantikine enzyme-linked immunosorbent assay (ELISA) kits specific to rat tumor necrosis factor α (TNF α, product number ab48910) and interleukin-6 (IL-6, product number Y11731A) were purchased from R&D Systems Inc. (Minneapolis, MN, U.S.). A terminal deoxynucleotidyl transferase-mediated dUTP nick end labeling (TUNEL) kit was purchased from Roche (product number 11684795910 Mannheim, Germany). Assay kits for malondialdehyde (MDA), superoxide dismutase (SOD), and lactate dehydrogenase (LDH) were purchased from Nanjing Jiancheng Bioengineering Institute (Nanjing, China).

### Cell culture and treatment

Primary cultures of neonatal rat ventricular cardiomyocytes were prepared by a method described previously [19]. Three days after the cells were seeded and the cultured cardiomyocytes were randomly divided into six groups: (1) Control group: Cardiomyocytes were continuously cultured for 4 h in DMEM medium. (2) LPS group: Cardiomyocytes were incubated for 4 h with LPS (25 μg/ml) alone. (3) GdCl_3_ group: Cardiomyocytes were cultured with 300 μM GdCl_3_ (activator of CaSR). (4) LPS + GdCl_3_ group: Cardiomyocytes were cultured with 25 μg/ml LPS and 300 μM GdCl_3_. (5) NPS2390 group: Cardiomyocytes were cultured with 10 μM NPS2390 (antagonist of CaSR). (6) LPS + NPS2390 group: Cardiomyocytes were cultured with 25 μg/ml LPS and 10 μM NPS2390. For controls, equivalent volumes of medium were added. Only cultures consisting of >95 % actin-positive cells as determined by counting 300 cells in three different fields were subjected to analysis.

### TUNEL staining

In accordance with the manufacturer’s protocol, apoptotic cells were assayed by TUNEL staining. The relative number of apoptotic cells was calculated as the ratio of the number of TUNEL-positive cells to the total number of cells, counted in three different random fields.

### TNF-α and IL-6 measurement

The concentration of TNF-α and IL-6 in the culture media were detected using an ELISA kit. The medium was collected and TNF-α levels were quantified using an ELISA assay kit specific to the rat TNF-α with a lower limit of detectability of 15 pg/ml. The lower detection limit of the IL-6 ELISA kit was 7.8 pg/ml.

### Measurement of MDA level, LDH activity, and SOD activity

The level of MDA, SOD, and LDH activity were measured using a commercial kit according to manufacturer’s instruction.

### Measurement of intracellular calcium

Cardiomyocytes were cultured in 96-well plates (the amount of cells was 5 × 10^5^/ml) and then loaded with 10 μM Fluo-3/AM for 60 min at 37 °C in the dark. They were then rinsed with Ca^2+^-free PBS three times to remove the extracellular Fluo-3/AM, and 200 μl of DMEM solution was added. Excitation was set at 488 nm, and emission was monitored at 530 nm. The loaded cardiomyocytes were stimulated with LPS alone (25 μg/ml), GdCl_3_ alone, NPS2390 alone, or LPS in combination with GdCl_3_ or NPS2390. The images of fluorescence, indicating [Ca^2+^]_i_, were recorded using laser confocal scanning microscope (Leica Corporation, Germany).

### Western blot analysis of CaSR

Total proteins of the neonatal rat myocytes were prepared according to manufacturer’s instructions. Protein concentration of the supernatant was determined using a Bradford protein assay with BSA as standard. Total proteins (20 μg) were electrophoresed through standard 10 % SDS-PAGE in Tris–glycine electrophoresis buffer, and blotted onto nitrocellulose membrane in transferring buffer at 100 V for 1 h in a water-cooled transfer apparatus. The membrane was blocked in a TBS-T buffer containing 5 % of skimmed milk at 37 °C for 1 h, and then incubated overnight at 4 °C with anti-CaSR antibody (1:2,500). Then, the membrane was washed three times with TBS-T and incubated with anti-IgG antibody conjugated with alkaline phosphatase diluted to 1:1,000 in TBS-T for 1 h at room temperature. Antibody–antigen complexes were detected using Western Blue ^®^Stabilized Substrate for alkaline phosphatase. The volume of the protein bands was quantified using a Bio-Rad Chemi Doc™ EQ densitometer and a Bio-Rad Quantity One software.

### Statistical analysis

All experiments were performed at least three times per determination. Data are expressed as mean ± SEM. Comparisons among the groups were performed using Kruskal–Wallis one-way ANOVA. Differences were considered significant at *P* value <0.05.

## Results

### Effects of CaSR on apoptosis among LPS-stimulated cardiomyocytes

**Fig. 1:**
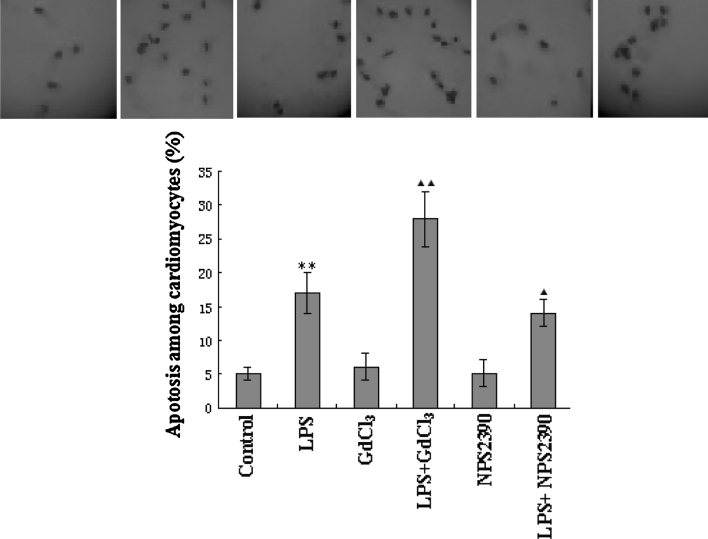
Rate of apoptotic cell (*n* = 8). First, 5 ± 1 % TUNEL positive nuclei were detected in control cells. In the LPS group, the % of apoptotic cells was 17 ± 3 %. After pretreatment with LPS and GdCl_3_, the relative number of apoptotic cells was 28 ± 4 %. Pretreatment with LPS and NPS2390 reduced the relative number of apoptotic cells to 14 ± 2 % (*P* < 0.05 vs LPS group). GdCl_3_ and NPS2390 alone had no effect on the apoptosis among cardiomyocytes. **P < 0.01 versus control group, ^▲▲^ *P* < 0.01 versus LPS group, ^▲^ *P* < 0.05 versus LPS group

Only 5 ± 1 % TUNEL positive nuclei were detected in control cells at the end of the experiment, and LPS significantly increased the percentage of apoptotic cells to 17 ± 3 % (*P* < 0.01 vs control). GdCl_3_ and NPS2390 alone had no effect on the apoptosis of cardiomyocytes. However, the pretreatment with LPS and GdCl_3_ increased the rate of apoptosis to 28 ± 4 % (*P* < 0.01 vs LPS group). NPS2390 treatment reduced the percentage of TUNEL-positive cells to 14 ± 2 % that of the LPS group (*P* < 0.05 vs LPS group) (Fig. 1).

### Effects of CaSR on LPS-induced release of TNF-α and IL-6 from neonatal rat cardiomyocytes

**Fig. 2:**
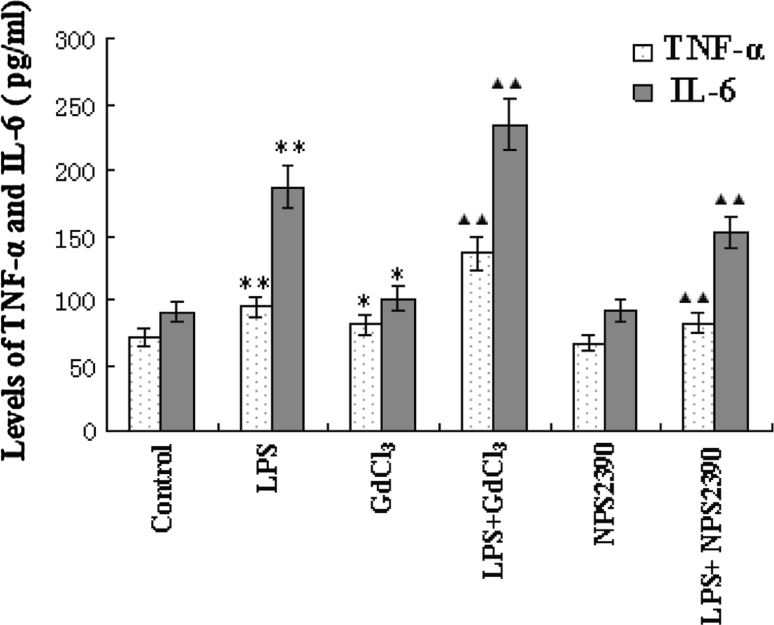
Release of TNF-α and IL-6 from cultured neonatal rat cardiomyocytes as detected using ELISA (*n* = 8). The cardiomyocytes were treated with GdCl_3_, NPS2390, and LPS either alone, with GdCl_3_, or with NPS2390 for 4 h. After incubation, the levels of TNF-α and IL-6 in the culture medium were measured using ELISA according to the manufacturer’s instructions. The levels of TNF-α and IL-6 in the media of cardiomyocytes were increased after exposure to LPS. GdCl_3_ was found to induce the release of TNF-α and IL-6 from cardiomyocytes. NPS2390 alone had no effect on the levels of TNF-α or IL-6. GdCl_3_ further elevated the rate of release of TNF-α and IL-6 induced by LPS. However, NPS2390 inhibited LPS-induced release of TNF-α and IL-6. *P < 0.05 versus control group, **P < 0.01 versus control group, ^▲▲^ *P* < 0.01 versus LPS group

Control cardiomyocytes released TNF-α and IL-6 at basal levels of 72.1 ± 6.4 and 91.1 ± 8.4 pg/ml, respectively. LPS significantly stimulated TNF-α and IL-6 release at levels of 95.9 ± 8.1 and 186.7 ± 16.5 pg/ml, respectively, which were higher than those in controls (*P* < 0.01). GdCl_3_ alone was found to increase TNF-α and IL-6 levels to 91.7 ± 7.4 and 116.7 ± 8.5 pg/ml, respectively (*P* < 0.05 vs control group). NPS2390 alone had no effect on TNF-α and IL-6 levels. LPS and GdCl_3_ were found to induce a significant increase in TNF-α, to a level of 136.7 ± 12.8 pg/ml, and IL-6 to 234.2 ± 19.3 pg/ml (*P* < 0.01 vs LPS group). However, NPS2390 inhibited CaSR and so significantly decreased LPS-induced release of TNF-α to 83.4 ± 7.3 pg/ml and IL-6 to 152.6 ± 11.3 pg/ml (*P* < 0.01 vs LPS group) (Fig. 2).

### Measurement of LDH, SOD, and MDA levels

In the LPS group, the levels of LDH and MDA were significantly higher than those in the control group, but SOD activity was significantly lower (*P* < 0.01). GdCl_3_ further elevated LDH and MDA levels and inhibited SOD activity (*P* < 0.01 vs LPS group). However, the presence of NPS2390 was found to decrease the concentrations of LDH and MDA and to increase SOD activity (*P* < 0.05 or *P* < 0.01 vs LPS group). Neither GdCl_3_ nor NPS2390 alone had any effect on LDH, SOD, or MDA levels (Table 1).

**Table 1:** Levels of LDH, MDA, and SOD activity in culture medium in different groups (*n* = 8)

Groups	LDH (U/ml)	SOD (U/ml) (nmol/ml)	MDA
Control	28.7 ± 2.6	38.1 ± 3.2	8.9 ± 0.7
LPS	58.7 ± 4.2**	31.4 ± 2.9**	18.8 ± 2.1**
GdCl_3_	31.6 ± 3.1	36.2 ± 2.4	10.2 ± 1.6
LPS + GdCl_3_	72.7 ± 6.3***	24.2 ± 2.0***	23.6 ± 3.4***
NPS2390	26.9 ± 2.2	35.3 ± 2.5	10.0 ± 1.7
LPS + NPS2390	49.7 ± 3.1***	34.1 ± 3.2*	14.5 ± 2.4***

** *P* < 0.01 versus control group, * *P* < 0.05, or *** *P* < 0.01 versus LPS group

### Influence of CaSR on the LPS-induced increase in [Ca^2+^]_i_ in cardiomyocytes

**Fig. 3:**
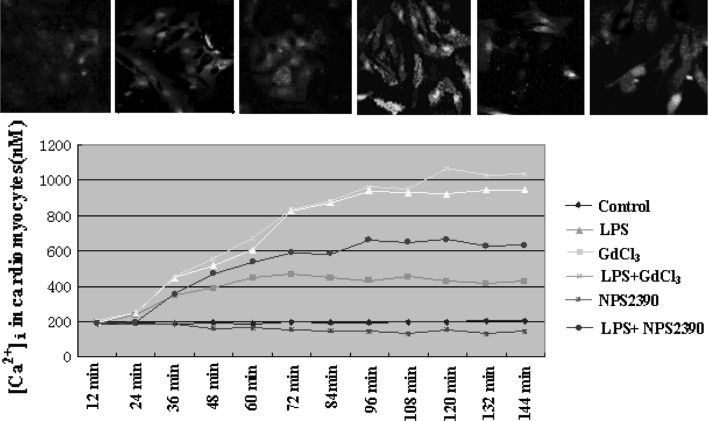
Intracellular calcium in cardiomyocytes (*n* = 8). Changes in the intensity of fluorescence of [Ca^2+^]_i_ were recorded continuously with a laser scanning confocal microscope under different treatment conditions. [Ca^2+^]_i_ was recorded for 144 min in total. GdCl_3_ further caused LPS-induced [Ca^2+^]_i_ increases in cultured neonatal rat cardiomyocytes. NPS2390 significantly inhibited the increases in [Ca^2+^]_i_ due to LPS

The effects of CaSR on the LPS-induced elevation in [Ca^2+^]_i_ in cardiomyocytes are shown in Fig. 3. LPS caused a remarkable increase in [Ca^2+^]_i_ within 144 min. Activation of CaSR also increased [Ca^2+^]_i_. In the presence of 300 μM GdCl_3_ and 25 μg/ml LPS, [Ca^2+^]_i_ in cardiomyocytes increased significantly. Pretreatment with 10 μM NPS2390 significantly inhibited the LPS-induced increases in [Ca^2+^]_i_ (Fig. 3).

### Effects of LPS and GdCl_3_ on CaSR expression

**Fig. 4:**
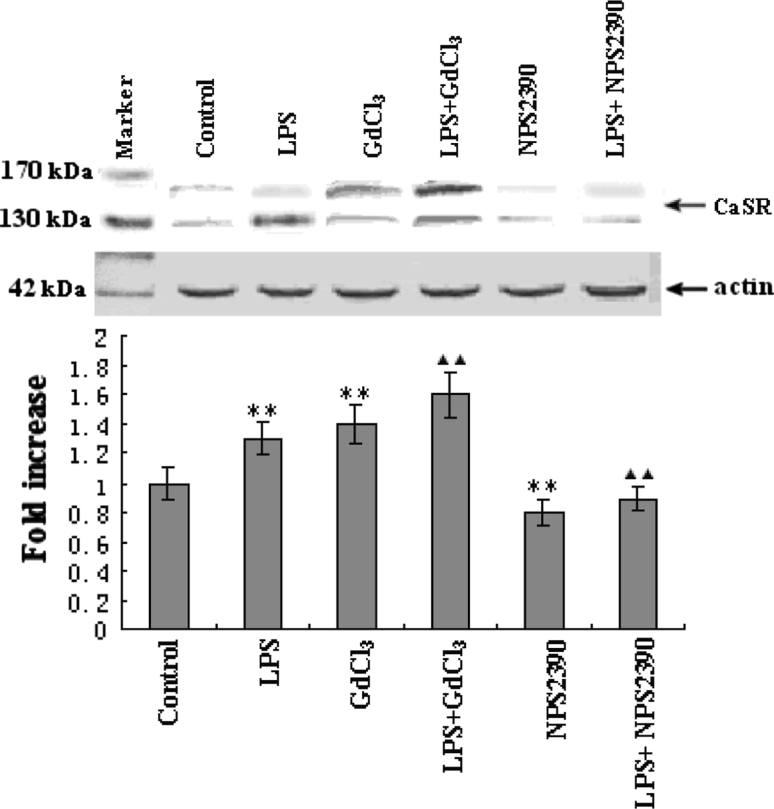
Expression of CaSR in cardiomyocytes as indicated by Western blot (*n* = 8). Basal level of CaSR was detected in the control group. This served as a baseline for comparison of fold increases in other groups. The expression of CaSR in the LPS group was higher than in the control group. GdCl_3_ alone was found to induce CaSR expression, but NPS2390 was found to inhibit CaSR expression. Pretreatment with GdCl_3_ further increased LPS-induced CaSR expression. However, NPS2390 decreased LPS-induced CaSR expression. Quantitation of Western blot analysis for CaSR is shown in the *lower panel*. The fold change values represent mean ± SEM. **P < 0.01 versus control group, ^▲▲^ *P* < 0.01 versus LPS group

The expression of CaSR was higher in the LPS group than that in the control group (*P* < 0.05 vs control). After incubation with LPS and GdCl_3_ for 4 h, the CaSR expression level increased further (*P* < 0.05 vs LPS group), but LPS and NPS2390 were found to reduce the level of expression of CaSR (*P* < 0.05 vs LPS group) (Fig. 4).

## Discussion

Calcium-sensing receptor is expressed in cardiac tissues. Increasing amounts of evidence indicate that CaSR may play a role in cardiomyocyte function. However, the role of CaSR in LPS-induced myocardial dysfunction has yet not been fully defined. LPS is a major structural component of Gram-negative bacteria, and a key mediator of the body’s response to infection [21, 22]. LPS induces expression of pro-inflammatory cytokines, such as TNF-α and IL-6, which are involved in the pathogenesis of sepsis and are also an early predictors of organ dysfunction [23–25]. TNF-α and IL-6 have been demonstrated to impair cardiac contractile function in intact animals, isolated hearts, and cardiomyocytes. In this way, plasma concentrations of both these factors are valuable predictors of the prognosis of these conditions.

Lipopolysaccharide induces TNF-α release in cardiomyocytes which may be a major local source of TNF-α in the myocardium during sepsis [26]. Blocking TNF-α reduces myocardial depression induced by endotoxemia [27]. In this way, the modulation of local myocardial TNF-α levels produced by cardiomyocytes may be of therapeutic significance in sepsis-induced myocardial dysfunction. To determine whether CaSR has any effect on TNF-α and IL-6 release during LPS-induced myocardial dysfunction, we measured the levels of TNF-α and IL-6. Our results showed that after LPS challenge, TNF-α and IL-6 releases were enhanced.

NPS2390 is a potent and selective noncompetitive group I metabotropic glutamate receptor (mGluR) antagonist [28]. Because CaSR is reported to share considerable structural similarity with mGluR1, NPS2390 has been used as a CaSR antagonist in previous studies [29]. In the present study, treatment with NPS2390 was found to decrease the amount of TNF-α and IL-6 released. However, the CaSR activator GdCl_3_ was found to promote LPS-stimulated TNF-α and IL-6 release in cardiomyocytes. This suggests that CaSR might lead to cell injury through upregulation of TNF-α and IL-6.

The TNF-α and IL-6 production induced by LPS is dependent on increases in [Ca^2+^]_i_ and in the activity of certain signal pathways in cardiomyocytes [30]. The elevation of [Ca^2+^]_i_ involves intracellular calcium release and calcium influx. High levels of intracellular calcium trigger cardiomyocyte injury. Wang et al. [11] have reported that, in isolated ventricular adult cardiomyocytes, increased extracellular calcium and gadolinium can induce a sustained, concentration-dependent increase in [Ca^2+^]_i_ through the CaSR–PLC–IP_3_ pathway. Our previous study has suggested that CaSR is involved in ischemia/reperfusion injury and apoptosis in neonatal rat ventricular cardiomyocytes through induction of calcium overload [18–20]. To determine whether CaSR is involved in calcium overload during LPS-induced myocardial dysfunction, alterations in intracellular calcium concentration after cellular exposure to LPS were observed. [Ca^2+^]_i_ was found to increase after exposure to LPS. The results showed that NPS2390, a CaSR antagonist, partially inhibited the increase in [Ca^2+^]_i_ induced by LPS. GdCl_3_, an activator of CaSR, enhanced the elevation of [Ca^2+^]_i_ in both normal and LPS-pretreated cardiomyocytes. These data suggest that CaSR may play a part in the LPS-induced elevation of [Ca^2+^]_i_ in cardiomyocytes.

Oxidative stress is increased in the myocardium after sepsis, and it plays significant roles in cardiac myocyte death and loss of cardiac function. The activity of SOD can indicate the cellular capability of scavenging and quenching free radicals. MDA, the degradation product of the oxygen-derived free radicals and lipid oxidation, interferes with the proteins, glucose, and nucleic acid metabolism, which can cause nucleic acid dysfunction and cellular injury. Cardiomyocyte apoptosis is another pathogenic mechanism underlying LPS injury. This article addressed the issue of whether CaSR plays a role in cardiomyocyte apoptosis induced by LPS. The results showed that CaSR significantly increased LDH and MDA levels, decreased SOD activity, and induced apoptosis among cardiomyocytes. CaSR expression also increased remarkably in response to LPS. These results suggested that activation of CaSR induced significant cardiomyocyte under LPS stimulation.

In the present study, NPS2390 partially prevented the LPS-stimulated increase in [Ca^2+^]_i_ and in turn decreased proinflammatory cytokine activation in cardiomyocytes. NPS2390 was found to inhibit apoptosis among cardiomyocytes induced by LPS, decrease the LDH and MDA levels, and increase SOD activity during endotoxemia.

The present study provides direct evidence demonstrating that CaSR is present in neonatal rat cardiomyocytes. CaSR was found to increase levels of intracellular calcium during LPS-induced heart injury and to induce proinflammatory cytokine activation. These findings may provide an explanation for the action of CaSR during LPS-induced heart injury.

In summary, the present study demonstrated that injury to the rat myocardium induced by LPS could be, at least in part, mediated by CaSR.
